# Activin Receptor-Like Kinase Receptors ALK5 and ALK1 Are Both Required for TGFβ-Induced Chondrogenic Differentiation of Human Bone Marrow-Derived Mesenchymal Stem Cells

**DOI:** 10.1371/journal.pone.0146124

**Published:** 2015-12-31

**Authors:** Laurie M. G. de Kroon, Roberto Narcisi, Esmeralda N. Blaney Davidson, Mairéad A. Cleary, Henk M. van Beuningen, Wendy J. L. M. Koevoet, Gerjo J. V. M. van Osch, Peter M. van der Kraan

**Affiliations:** 1 Department of Rheumatology, Experimental Rheumatology, Radboud University Medical Center, Nijmegen, the Netherlands; 2 Department of Orthopedics, Erasmus MC University Medical Center, Rotterdam, the Netherlands; 3 Section of Veterinary Clinical Sciences, School of Veterinary Medicine, University College Dublin, Dublin, Ireland; 4 Department of Otorhinolaryngology, Erasmus MC University Medical Center, Rotterdam, the Netherlands; Stony Brook University, UNITED STATES

## Abstract

**Introduction:**

Bone marrow-derived mesenchymal stem cells (BMSCs) are promising for cartilage regeneration because BMSCs can differentiate into cartilage tissue-producing chondrocytes. Transforming Growth Factor β (TGFβ) is crucial for inducing chondrogenic differentiation of BMSCs and is known to signal via Activin receptor-Like Kinase (ALK) receptors ALK5 and ALK1. Since the specific role of these two TGFβ receptors in chondrogenesis is unknown, we investigated whether ALK5 and ALK1 are expressed in BMSCs and whether both receptors are required for chondrogenic differentiation of BMSCs.

**Materials & Methods:**

ALK5 and ALK1 gene expression in human BMSCs was determined with RT-qPCR. To induce chondrogenesis, human BMSCs were pellet-cultured in serum-free chondrogenic medium containing TGFβ1. Chondrogenesis was evaluated by aggrecan and collagen type IIα1 RT-qPCR analysis, and histological stainings of proteoglycans and collagen type II. To overexpress constitutively active (ca) receptors, BMSCs were transduced either with caALK5 or caALK1. Expression of ALK5 and ALK1 was downregulated by transducing BMSCs with shRNA against ALK5 or ALK1.

**Results:**

ALK5 and ALK1 were expressed in *in vitro*-expanded as well as in pellet-cultured BMSCs from five donors, but mRNA levels of both TGFβ receptors did not clearly associate with chondrogenic induction. TGFβ increased ALK5 and decreased ALK1 gene expression in chondrogenically differentiating BMSC pellets. Neither caALK5 nor caALK1 overexpression induced cartilage matrix formation as efficient as that induced by TGFβ. Moreover, short hairpin-mediated downregulation of either ALK5 or ALK1 resulted in a strong inhibition of TGFβ-induced chondrogenesis.

**Conclusion:**

ALK5 as well as ALK1 are required for TGFβ-induced chondrogenic differentiation of BMSCs, and TGFβ not only directly induces chondrogenesis, but also modulates ALK5 and ALK1 receptor signaling in BMSCs. These results imply that optimizing cartilage formation by mesenchymal stem cells will depend on activation of both receptors.

## Introduction

Articular cartilage is a connective tissue covering bone surfaces within synovial joints and its main functions are distributing forces and preventing friction between moving bones. Chondrocytes are the only cell population present in articular cartilage, and they are responsible for the production, maintenance and organization of cartilage matrix [1]. Once articular cartilage becomes damaged by an injury or joint disease, it is not regenerated. In the long run, articular cartilage defects get worse and patients suffer from joint pain and dysfunction [2]. Therefore, innovative treatments to regenerate cartilage tissue are highly necessary. However, to date, a procedure in which articular cartilage tissue is formed closely resembling native tissue’s structure and properties still needs to be developed [3, 4].

To heal damaged cartilage, bone marrow-derived mesenchymal stem cells (BMSCs) are promising due to their capacity to differentiate into chondrocytes [5–11]. Unfortunately, thus far, results from BMSC-based therapies have been suboptimal due to variable levels of cartilage tissue formation and poor functional properties of the repair tissue formed [12–15]. To solve these issues regarding the functionality of BMSC-derived cartilage, we need to better understand the molecular mechanisms that control chondrogenic differentiation of BMSCs. Ultimately, this might lead to formation of cartilage tissue which yields similar properties as native cartilage and restores joint functionality.

Transforming growth factor β (TGFβ), originally described as Cartilage-Inducing Factor [16], is required for chondrogenic differentiation of human BMSCs [5–9, 11]. TGFβ signals via type I and II serine/threonine kinase receptors [17]. Upon binding of TGFβ ligands to type II receptors, heteromeric complexes are formed with type I receptors [18–20]. Within a complex, type II receptors phosphorylate the kinase domain of type I activin receptor-like kinase receptors (ALKs) which then recruit and phosphorylate SMAD proteins. Phosphorylated SMAD proteins translocate to the nucleus, where they associate with transcriptional co-activators and co-repressors to modulate target gene expression in a cell type-specific manner [21–23].

Although in most cell types TGFβ signaling is conveyed through ALK5, multiple studies have identified ALK1 as an alternative type I receptor for TGFβ [24–30]. ALK5 is known to phosphorylate SMAD2 and SMAD3 proteins (SMAD2/3), and ALK1 phosphorylates SMAD1, SMAD5 and SMAD8 proteins (SMAD1/5/8) [22, 31]. Previously, our group demonstrated that both SMAD pathways are important in chondrogenic differentiation of BMSCs as blocking TGFβ-induced phosphorylation of either SMAD2/3 or SMAD1/5/8 with chemical inhibitors prevented BMSC chondrogenesis [32]. However, the specific role of ALK5 and ALK1 receptors in chondrogenic induction of BMSCs with TGFβ remains unknown. As TGFβ is crucial for BMSC chondrogenesis and is known to activate ALK5 as well as ALK1 receptors, our aim was to elucidate whether ALK5 and ALK1 are required for TGFβ-induced chondrogenic differentiation of human BMSCs.

## Materials and Methods

### Cell isolation and expansion

#### Human fetal bone marrow-derived mesenchymal stem cells

Primary human fetal BMSCs from one donor (donor F1) were purchased from ScienCell Research Laboratories (Carlsbad, CA, USA). Cells were culture-expanded in Mesenchymal Stem Cell Growth Medium (Lonza, Basel, Switzerland) supplemented with 1% Penicillin-Streptomycin-Glutamine (Gibco/Thermo Fisher Scientific, Waltham, MA, USA) until subconfluency and stored in liquid nitrogen at passage 5. Per experiment, fetal BMSCs were defrosted and expanded for another 2 to 4 passages.

#### Human adult bone marrow-derived mesenchymal stem cells

After obtaining written informed consent from patients (n = 5), bone marrow aspirates were collected during total hip arthroplasty for osteoarthritis. This procedure was approved by the medical ethical committees of the Erasmus Medical Center, Rotterdam (#METC 2004–142) and Albert Schweitzer Hospital, Dordrecht (#METC 2011.07). Primary human adult BMSCs were isolated from the marrow aspirate from 5 donors (Donor A1 –A5) by plastic adherence. Adult BMSCs were seeded in α-MEM (Gibco) containing 10% pre-selected fetal calf serum (FCS; Lonza), 50 μg/mL gentamicine, 1.5 μg/mL fungizone (both from Gibco), 10^−4^ M L-Ascorbic acid 2-phosphate (Sigma-Aldrich, St. Louis, MO, USA) and 1 ng/mL Fibroblast Growth Factor-2 (AbD Serotec, Kidlington, United Kingdom). Non-adherent cells were removed after 24 hours and adherent cells were further culture-expanded until subconfluency. Experiments were performed using adult BMSCs in passage 2 to 4.

### Chondrogenic differentiation of human adult and fetal BMSCs

BMSCs were centrifuged at 200 x g for 8 minutes and the obtained cell pellets (0.2×10^6^ cells/pellet) were cultured for up to 21 days in 0.5 mL serum-free chondrogenic medium which consisted of DMEM-high glucose-GlutaMAX™, 1.5 mg/mL fungizone, 50 mg/mL gentamicin, 1.0 mg/mL sodium pyruvate (all from Gibco), 6.25 μg/mL insulin, 6.25 μg/mL transferrin, 6.25 ng/mL selenious acid, 5.35 μg/mL linoleic acid, 1.25 mg/mL bovine serum albumin, 0.4 mg/mL L-proline, 10^−4^ M L-Ascorbic acid 2-phosphate, 10^−7^ M dexamethasone (all from Sigma-Aldrich) and 10 ng/mL TGFβ1 (adult BMSCs; R&D Systems, Minneapolis, MN, USA and fetal BMSCs; Biolegend, San Diego, CA, USA). In parallel, pellets were cultured in chondrogenic medium without TGFβ1 supplementation in order to determine the effects of TGFβ on ALK5 and ALK1 expression. This was done with cells from donors A1 and F1 because only from these two donors a sufficient number of cells was available. Medium was renewed 2 or 3 times a week. Triplicate pellets were harvested for mRNA expression analysis and histological examination.

### Overexpression of caALK5 and caALK1

Adenoviruses for constitutively active (ca) caALK5, caALK1 or LacZ were kindly provided by Dr. P. ten Dijke (Leiden University Medical Center, the Netherlands), and their preparation was described by Nakao *et al*. and Itoh *et al*. [22, 33].

Viral overexpressions were performed in BMSCs from fetal donor F1 because a high number of cells was available. Moreover, BMSCs from this donor chondrogenically differentiated within 7 days which is preferred to reliably test the effect of viral transductions, as adenoviruses cannot integrate into the cell’s genome. Furthermore, donor F1 tested negative for HIV which is an important requirement for lentiviral transductions. Due to ethical concerns, the HIV-status could not be tested in donors A1-A5.

Prior to cell pellet preparation, fetal BMSCs cultured in monolayer were transduced in serum-free DMEM-high glucose-GlutaMAX™ (Gibco) with adenovirus overexpressing caALK5 or caALK1 or LacZ (control) at a multiplicity of infection (MOI) of 150 pfu/cell for 3 hours. Cells were washed and pellet-cultured in serum-free chondrogenic medium for 7 days. In the presence of TGFβ, fetal BMSC pellets chondrogenically differentiated within 7 days as determined by *ACAN* and *COL2A1* RT-qPCR analysis and histological examination of proteoglycans and collagen type II. To test whether constitutively active ALK5 or ALK1 receptor signaling induced chondrogenic differentiation, BMSCs either transduced with caALK5 or caALK1 were not stimulated with TGFβ and LacZ-transduced BMSCs were stimulated with TGFβ.

To verify the overexpression of caALK5 and caALK1 in BMSC pellets one day after transduction, mRNA levels of ALK5 (= *TGFBR1*) and ALK1 (= *ACVRL1*) were determined by RT-qPCR. In order to confirm that caALK5 induced SMAD2 phosphorylation (pSMAD2) and that caALK1 induced SMAD1/5/8 phosphorylation (pSMAD1/5/8), expression of pSMAD2 and pSMAD1/5/8 proteins were analyzed by Western blot in one day-cultured BMSC pellets transduced with caALK5, caALK1 or LacZ. Expression of pSMAD2 and pSMAD1/5/8 was compared between LacZ-transduced pellets (not exposed to TGFβ) and pellets transduced either with caALK5 or caALK1.

### shRNA-mediated downregulation of ALK5 and ALK1 expression

MISSION® TRC-Hs1.5 shRNA clones (Sigma-Aldrich) targeting ALK5 (TRCN0000039773) or ALK1 (TRCN0000000355) and Empty Vector Control (SHC001) constructed in the pLKO.1-Puro plasmid vector were kindly provided as purified plasmid DNA by Dr. P. ten Dijke (Leiden University Medical Center, the Netherlands). Lentiviruses were packaged with one of the aforementioned pLKO.1-puro plasmid vectors as described below.

HEK293T cells (ATCC, Manassas, VA, USA) were co-transduced with 42.7 μg pLKO.1-puro plasmid, 31.8 μg *gag/pol* packaging plasmid (pMDL-g/p-RRE), 10.7 μg *rev* expression plasmid (RSV-REV) and 15.0 μg VSV-G expression plasmid (pHIT-G) by calcium phosphate precipitation for 16 hours. Co-transductions were carried out in DMEM-high glucose-GlutaMAX™ (Gibco) containing 10% FCS (Perbio Science, Erembodegem, Belgium), 0.01 mM cholesterol (Sigma-Aldrich) and 1% pyruvate (Gibco). This medium (= supernatant) was renewed 16 hours after transduction, and 1% Penicillin-Streptomycin-Glutamine (Gibco) was added. After 24 and 48 hours from the medium renewal, the supernatant was collected and filtered through a 0.45 μm pore polyvinylidene fluoride Durapore filter (Millipore, Bedford, MA, USA). To concentrate lentiviral particles, 20% sucrose (Sigma-Aldrich) in PBS was pipetted under the supernatant and centrifuged at 134,350 x g for 2 hours (Sorvall WX80+, Thermo Fisher Scientific). After re-suspending the pellet in 210 μL sterile PBS, the concentration of lentiviral particles was determined according to the INNOTEST® HIV p24 Antigen monoclonal antibody assay (Fujirebio Europe, Gent, Belgium) and lentivirus titers were expressed as pg of p24/μL.

Fetal BMSCs, cultured in monolayer, were transduced for 24 hours with 1 pg p24 (lentivirus) per cell of either ALK5-shRNA, ALK1-shRNA or empty vector control in Mesenchymal Stem Cell Growth Medium (Lonza) supplemented with 1% Penicillin-Streptomycin-Glutamine (Gibco) and 100 μg/mL protamine sulfate (Sigma-Aldrich). Following lentiviral transduction, cells were washed, trypsinized (0.25% trypsin; Gibco) and pellet-cultured in serum-free chondrogenic medium containing TGFβ1. After 7 days, chondrogenic induction was evaluated by determining expression of cartilage-specific genes *(ACAN* and *COL2A1)* and proteins (proteoglycan and collagen type II). In pellets cultured for 1 or 7 days, *TGFBR1* and *ACVRL1* mRNA levels were analyzed by RT-qPCR to evaluate the efficiency of shRNA-mediated downregulation of ALK5 and ALK1.

### RNA extraction and real-time quantitative polymerase chain reaction

Total cellular RNA was extracted from fetal BMSCs with TRI Reagent® (Sigma-Aldrich) according to manufacturer’s protocol and contaminating DNA was removed by DNAse treatment (Invitrogen/Thermo Fisher Scientific). Adult BMSCs were homogenized in RNA-Bee (Tel-Test, Friendswood, TX, USA) and RNA was further purified using an RNeasy MicroKit (Qiagen, Hilden, Germany) with on-column DNA digestion. The concentration and purity of isolated RNA were measured on a NanoDrop® spectrophotometer (Isogen Life Science, Utrecht, the Netherlands). 500ng of total RNA was converted into cDNA using Reverse Transcriptase (Invitrogen) according to manufacturer’s instructions. Real-Time Quantitative Polymerase Chain Reaction (RT-qPCR) measurements were performed on a StepOnePlus™ Real-Time PCR System using SYBR Green Master mix (both from Applied Biosystems/Thermo Fisher Scientific). Primers are listed in Table 1.

**Table 1:** List of primers used for RT-qPCR.

	Primer sequences		Details	
Gene	Forward primer	Reverse primer	Product	Slope
***RPS27a***	TGGCTGTCCTGAAATATTATAAGGT	CCCCAGCACCACATTCATCA	90bp	3.4
***TGFBR1* (ALK5)**	CGACGGCGTTACAGTGTTTCT	CCCATCTGTCACACAAGTAAA	65bp	3.3
***ACVRL1* (ALK1)**	CCATCGTGAATGGCATCGT	GGTCATTGGGCACCACATC	63bp	3.2
***ACAN***	GCCTGCGCTCCAATGACT	ATGGAACACGATGCCTTTCAC	104bp	3.3
***COL2A1***	CACGTACACTGCCCTGAAGGA	CGATAACAGTCTTGCCCCACTT	65bp	3.3

### Gene expression analysis

The Ct value of each primer was determined at a fixed threshold level of fluorescence and the amplification efficiency of all primers was between 90% and 110% (Table 1). Gene expression was normalized to expression of ribosomal protein 27a (*RPS27a*), which was stably expressed between conditions and over time. Normalized gene expression levels in control conditions were set as 100%. –ΔCt values were used for statistical analyses.

### Histology

Pellets were fixed in 4% formalin in PBS for 24 hours followed by paraffin embedding. Sections (6 μm thick) were mounted on Super Frost glass slides (Thermo Fisher Scientific). Proteoglycans were stained with 0.1% aqueous Safranin O and sections were counterstained with 0.1% aqueous Fast Green (both from Brunschwig Chemie, Amsterdam, the Netherlands). To stain collagen type II using immunohistochemistry, sections were pre-treated with 0.1% pronase (Sigma-Aldrich) in PBS at 37°C for 30 minutes followed by 30 minutes-incubation with 1% hyaluronidase (Sigma-Aldrich) in PBS at 37°C for antigen retrieval. Sections were rinsed with PBS and non-specific binding sites were blocked with 10% normal goat serum (SouthernBiotech, Birmingham, AL, USA) in PBS containing 1% BSA (PBS-1%BSA). After 30 minutes, sections were incubated either with 0.4 μg/mL anti-collagen II primary antibody (mouse monoclonal IgG1, raised against chicken collagen type II; II-II6B3 cell culture supernatant, Developmental Studies Hybridoma Bank, Iowa City, IA, USA) in PBS-1%BSA or incubated with 0.4 μg/mL mouse-IgG1 as negative control (#X0931, Dako, Glostrup, Denmark) in PBS-1%BSA at room temperature for 60 minutes. Subsequently, sections were rinsed with PBS and anti-collagen type II antibody was detected using alkaline phosphatase-conjugated secondary antibody kit (1:50 in PBS-1%BSA; #HK-321-UK, Biogenex, San Ramon, CA, USA) according to manufacturer’s protocol. Alkaline phosphatase activity, representing presence of collagen II protein, stained magenta after incubating sections with New Fuchsine substrate (Chroma, Köngen, Germany). Finally, sections were counterstained with hematoxylin (Sigma-Aldrich), air-dried and mounted with VectaMount™ (Vector Laboratories, Burlingame, CA, USA).

### Western blot analysis

Phosphorylated SMAD2 (pSMAD2) and phosphorylated SMAD1/5/8 (pSMAD1/5/8) protein expression was analyzed by Western blot. Total proteins were isolated from fetal BMSC pellets using Cell Lysis Buffer (Cell Signaling Technology, Danvers, MA, USA) with 1% protease inhibitor (Roche Diagnostics, Basel, Switzerland). Lysates were sonicated on ice using a Bioruptor® (Diagenode, Denville, NJ, USA). Sonified samples were centrifuged at 4°C at 16,000 x g for 15 minutes. In supernatant, the protein concentration was determined using the bicinchoninic acid assay (Thermo Fisher Scientific). 14 μg of protein lysate was loaded on a 10% bisacrylamide gel for SDS-PAGE. Hereafter, proteins were transferred to a nitrocellulose membrane (GE Healthcare, Little Chalfont, United Kingdom) by wet transfer in Towbin buffer (4°C) at 275 mA for 2 hours. Membranes were blocked with 5% non-fat dry milk (pSMAD2) or with 5% BSA (pSMAD1/5/8) in 0.1% Tween in Tris-buffererd saline (TBST). Subsequently, membranes were incubated overnight at 4°C with either rabbit-anti-pSMAD2 (1:1000; #3101L, Cell Signaling Technology) or rabbit-anti-p-Smad1/5/8 (1:1000; #9511L, Cell Signaling Technology) in 5% or 2% non-fat dry milk in TBST, respectively. After washing in TBST, membranes were incubated with anti-rabbit horseradish peroxidase (HPR)-linked secondary antibody (1:1500; #P0448, Dako) in TBST containing 5% non-fat dry milk (pSMAD2) or 2% non-fat dry milk (pSMAD1/5/8) at room temperature for 1 hour. Proteins were visualized by detection of the HRP-signal using enhanced chemiluminescence (ECL) Prime Western Blotting Detection Reagent kit (GE Healthcare) following manufacturer’s protocol.

To assess equal protein loading on pSMAD-stained membranes, GAPDH expression was analyzed. Membranes were blocked with Odyssey® Infrared Imaging System Blocking Buffer (LI-COR, Lincoln, NE, USA) for 1 hour, followed by incubation with mouse-anti-GAPDH antibody (1:10,000; #G8795, Sigma-Aldrich) in Odyssey blocking buffer at room temperature for 1 hour. After washing with PBS containing 0.1% Tween (PBST), membranes were incubated with donkey-anti-Mouse antibody conjugated with IRDye® 800CW (LI-COR) at room temperature for 30 minutes. GAPDH expression was detected using an Odyssey CLx Infrared Imaging System (LI-COR) and signal intensity was quantified with Image Studio™ Lite (Version 3.1, LI-COR) following software instructions.

### Data analysis

Quantitative data are represented as mean ± standard deviation. Statistical analyses were performed using GraphPad Prism version 5.03 for Windows (GraphPad Software, San Diego, CA, USA). The normality and variance were evaluated by the Shapiro-Wilk test and Levene’s Test of Homogeneity. Normally distributed data with equal variance were analyzed by an independent student’s t test or one-way ANOVA with Bonferroni post hoc test. Level of significance was set at p<0.05.

## Results

### Levels of ALK5 and ALK1 expression in BMSCs are not associated with chondrogenic differentiation capacity

**Fig 1:**
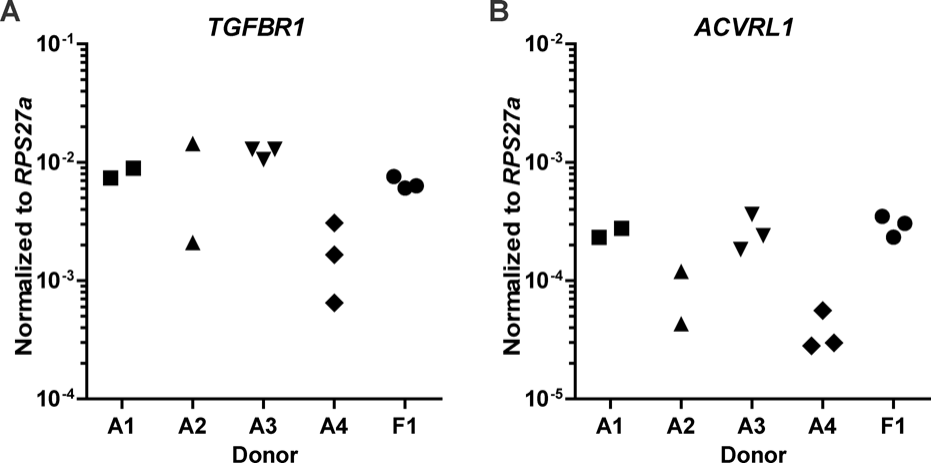
ALK5 and ALK1 are expressed in human bone marrow-derived mesenchymal stem cells (BMSCs). In human BMSCs, obtained from four adult donors (A1 –A4) and one fetal donor (F1), gene expression levels of ALK5 *(= TGFBR1)* **(A)** and ALK1 *(= ACVRL1)* **(B)** were analyzed using RT-qPCR. Gene expression was normalized to reference gene *RPS27a*. Each data point represents one measurement.

Transforming Growth Factor-β is known to signal either via ALK5 or ALK1 receptors, which intracellularly activate SMAD2/3 and SMAD1/5/8 signaling respectively. We first confirmed gene expression of ALK5 *(TGFBR1)* and ALK1 *(ACVRL1)* in *in vitro*-expanded BMSCs (n = 5) and observed that their expression levels varied between donors (Fig 1).

**Fig 2:**
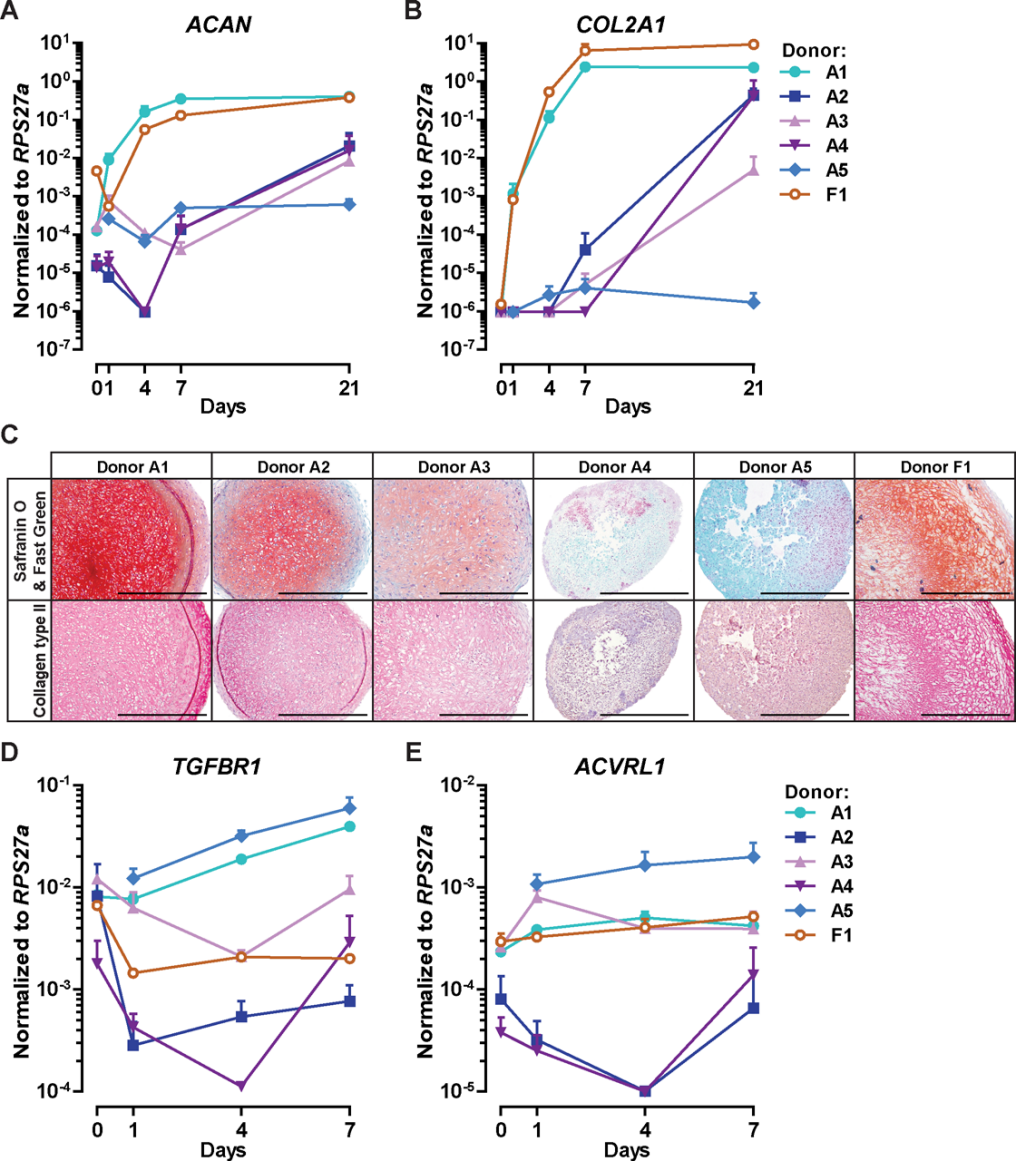
Gene expression levels of ALK5 *(TGFBR1)* and ALK1 *(ACVRL1)* are not associated with the chondrogenic capacity of human BMSCs. To induce chondrogenesis, BMSCs (passage 2–4) from adult donors A1 –A5 and fetal donor F1 were pellet-cultured in chondrogenic medium and stimulated with TGFβ. Cartilage-specific gene expression of aggrecan *(ACAN)* **(A)** and collagen type IIα1 (*COL2A1)* **(B)** was determined in BMSCs cultured for 0, 1, 4, 7 and 21 days using RT-qPCR. Cartilage-specific protein depositions of proteoglycans **(C; upper panel)** and collagen type II **(C; lower panel)** were stained in sections of 21 days-cultured pellets. To stain proteoglycans, sections were stained with Safranin O (red/orange) and Fast Green (blue) was used as counterstaining. Collagen type II was stained immunohistochemically (pink) and hematoxylin (purple) was used as counterstaining. Representative images of consecutive pellet sections per donor are shown and the scale bar represents 500 μm. *TGFBR1* **(D)** and *ACVRL1* **(E)** expression levels were measured in BMSC monolayer (day 0) and pellets cultured for 1, 4 and 7 days. Gene expression was normalized to reference gene *RPS27a*. Data points represent mean ± SD of triplicate BMSC pellets per donor per time point.

We next investigated whether the mRNA levels of ALK5 and ALK1 at onset of chondrogenic differentiation (first 7 days of pellet culture) were related to the chondrogenic capacity of BMSCs at day 21. Moreover, gene expression of aggrecan (*ACAN*; Fig 2A) and collagen type IIα1 (*COL2A1*; Fig 2B) was analyzed during onset of chondrogenesis. The overall chondrogenic capacity of each donor was determined by histological examination of proteoglycans and collagen type II deposition (Fig 2C) in pellets cultured for 21 days. Chondrogenic markers (Fig 2C) were highest expressed in pellets from donors A1, A2, A3 and F1 (defined as ‘highly chondrogenic’) and lowest in donors A4 and A5 (defined as ‘poorly chondrogenic’). At onset of chondrogenesis, ALK5 gene *(TGFBR1)* expression pattern was similar in donors A1 and A5, with a high expression at the start of culture and an increase over time (Fig 2D). The other 4 donors displayed lower *TGFBR1* expression than donors A1 and A5 and stable or decreasing expression over the 7 days of culture (Fig 2D). ALK1 mRNA *(ACVRL1)* levels remained rather stable over time in donors A1, A3, A5 and F1, while in donors A2 and A4 we generally observed a lower expression compared to the other donors (Fig 2E). Combined, these data indicate that ALK5 and ALK1 are expressed in human BMSCs, but with no clear association between mRNA levels of both TGFβ receptors and the chondrogenic capacity of BMSCs.

### TGFβ increases ALK5 and decreases ALK1 gene expression in BMSCs

As an increase in TGFβ levels and in the number of receptors might result in enhanced sensitivity of cells to TGFβ [34], we investigated whether TGFβ modulated *TGFBR1* (ALK5) and *ACVRL1* (ALK1) expression in BMSC pellets in donors A1 and F1 cultured for 1, 4 or 7 days. Compared to unstimulated pellets, TGFβ significantly induced *TGFBR1* expression in pellets from donor A1 and donor F1 at day 1, 4, and 7 (Table 2). More specifically, TGFβ enhanced transcript levels of *TGFBR1* in donor F1 about 2-fold at all time points measured, whereas in donor A1 TGFβ continuously increased *TGFBR1* mRNA levels over time; 2.55-fold at day 1, 3.31-fold at day 4 and 7.58-fold at day 7. Contrary to *TGFBR1* expression, *ACVRL1* appeared to be significantly downregulated by TGFβ in both donors tested (Table 2). The inhibiting effect of TGFβ on *ACVRL1* expression was more pronounced on day 4 than on day 1 in donor A1 (from -1.93-fold at day 1 to -3.30-fold at day 4) as well as in donor F1 (from -2.38-fold at day 1 to -3.32-fold at day 4). At day 7, *ACVRL1* mRNA levels were stronger decreased by TGFβ in donor A1 (-3.98-fold) than in donor F1 (-2.72-fold). Altogether, our data show that TGFβ regulates gene expression of its ALK5 and ALK1 receptors in chondrogenically differentiating BMSCs by enhancing *TGFBR1* and decreasing *ACVRL1* expression.

**Table 2:** TGFβ increases *TGFBR1* (ALK5) and decreases *ACVRL1* (ALK1) in human BMSC pellets.

	*TGFBR1* fold change (Mean ± SD)				*ACVRL1* fold change (Mean ± SD)			
Day	Donor A1	P-value	Donor F1	P-value	Donor A1	P-value	Donor F1	P-value
**1**	2.55 ± 0.39	0.010	1.98 ± 0.22	0.001	-1.93 ± 0.21	0.040	-2.38 ± 0.30	0.001
**4**	3.31 ± 0.27	0.001	2.24 ± 0.12	˂0.001	-3.30 ± 0.51	0.003	-3.32 ± 0.74	0.001
**7**	7.58 ± 0.26	˂0.001	2.34 ± 0.19	0.005	-3.98 ± 1.06	0.001	-2.72 ± 0.20	˂0.001

The effect of TGFβ on *TGFBR1* and *ACVRL1* was evaluated in BMSC pellets from donor A1 and donor F1 cultured in chondrogenic medium with TGFβ or without (= unstimulated) for 1, 4 or 7 days. Gene expression was normalized to reference gene *RPS27a*. Data are expressed as fold change relative to unstimulated pellets and the mean ± SD of triplicate BMSC pellets per donor per time point are shown.

### Overexpression of caALK5 or caALK1 receptors does not induce chondrogenic differentiation as efficiently as TGFβ

Since it was unknown whether TGFβ induces chondrogenesis through activation of its ALK5 or ALK1 receptor, we tested whether overexpression of either constitutively active (ca) caALK5 or caALK1 receptors induced chondrogenic differentiation of BMSCs.

**Fig 3:**
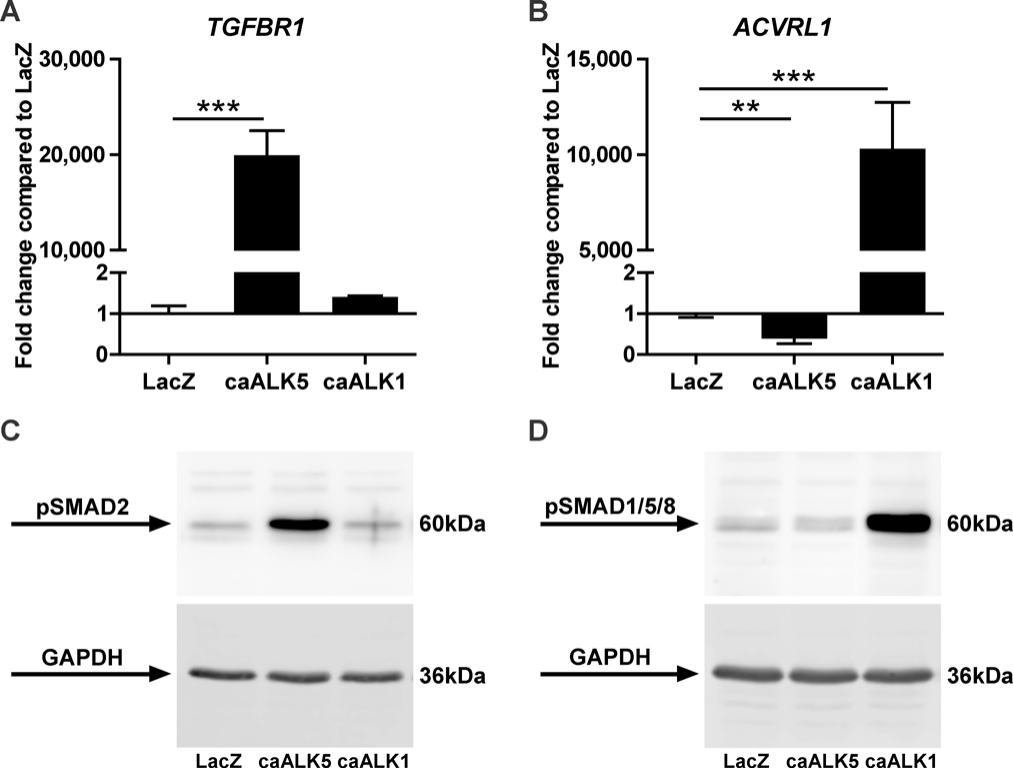
BMSCs overexpressing either constitutively active (ca) caALK5 or caALK1 express enhanced levels of phosphorylated SMAD2/3 and SMAD1/5/8 proteins, respectively. Human fetal BMSCs (donor F1) were transduced with adenoviral caALK5, caALK1 or LacZ as control and pellet-cultured in chondrogenic medium for 1 day. Transduction efficiency of caALK5 and caALK1 was evaluated after gene expression analysis of *TGFBR1* **(A)** and *ACVRL1* **(B)**. Gene expression was normalized to reference gene *RPS27a* and data are expressed as fold change relative to normalized gene levels in LacZ-transduced BMSCs. Bars represent mean ± SD from triplicate pellets of 1 representative experiment (out of 3), **p<0.01; ***p<0.001. Constitutively active receptor signaling of ALK5 and ALK1 was evaluated by Western blot analyses of phosphorylated SMAD (pSMAD) proteins; pSMAD2/3 **(C)** and pSMAD1/5/8 **(D)** using GAPDH as loading control.

In one day-cultured pellets we confirmed overexpression of *TGFBR1* following caALK5 transduction (Fig 3A) and *ACVRL1* overexpression following caALK1 transduction (Fig 3B). Whereas overexpression of caALK1 did not change *TGFBR1* expression (Fig 3A), caALK5 overexpression reduced mRNA levels of *ACVRL1* (Fig 3B). We also verified that caALK5 and caALK1 receptors phosphorylated their downstream signaling proteins SMAD2/3 (Fig 3C) and SMAD1/5/8 (Fig 3D) without prior activation by TGFβ and dimerization with type II receptors.

**Fig 4:**
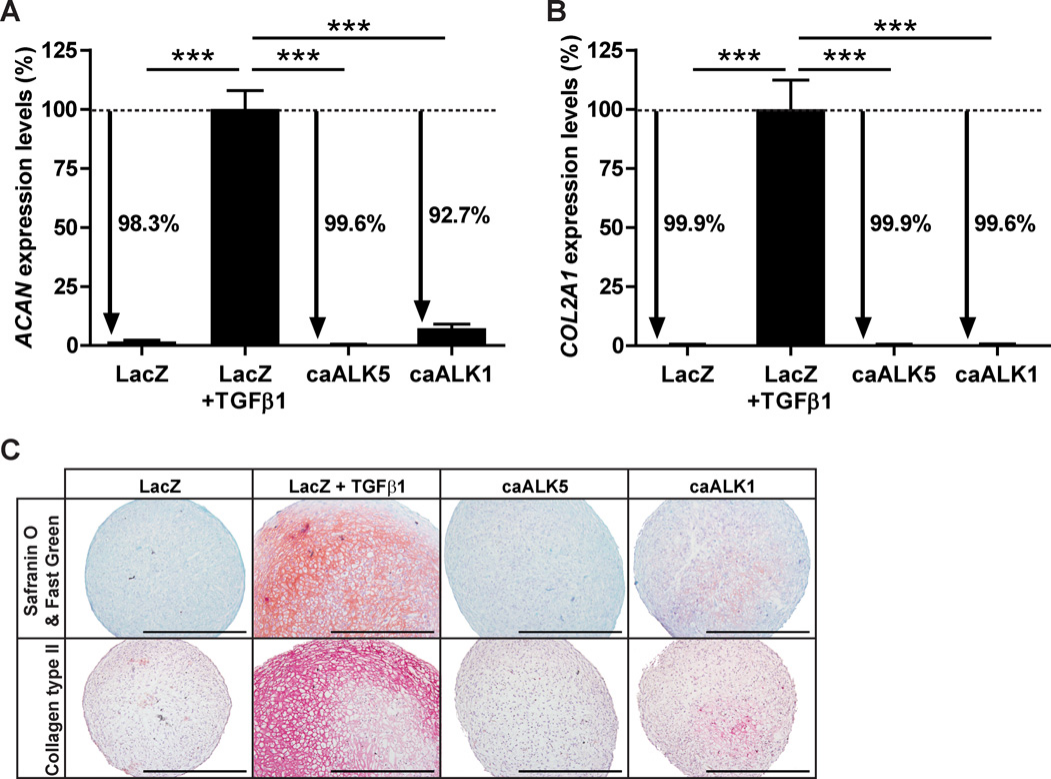
Constitutively active (ca) caALK5 and caALK1 receptors do not induce BMSC chondrogenesis as efficiently as TGFβ does. Human fetal BMSCs (donor F1) transduced with adenoviral caALK5, caALK1 or LacZ as control were pellet-cultured in chondrogenic medium for 7 days. Only LacZ-transduced pellets were exposed to TGFβ to compare if either caALK5 or caALK1 induced chondrogenesis as efficiently as induced by TGFβ. Chondrogenesis was evaluated by cartilage-specific gene expression analysis of *ACAN* **(A)** and *COL2A1* **(B)** and by staining proteoglycans with Safranin O/Fast Green **(C; upper panel)** and collagen type II with immunohistochemistry **(C; lower panel)**. Representative images of consecutive pellet sections per condition are shown and the scale bar represents 500 μm. Gene expression was normalized to reference gene *RPS27a* and data are expressed as % relative to normalized gene levels in LacZ-transduced pellets stimulated with TGFβ. Bars represent mean ± SD from quadruplet pellets of 1 representative experiment (out of 3), ***p<0.001.

Next, we determined whether overexpression of either caALK5 or caALK1 induced chondrogenic differentiation of human fetal BMSCs similar to chondrogenesis induced by TGFβ. Chondrogenic differentiation was evaluated by comparing expression of cartilage matrix-specific genes *(ACAN* and *COL2A1)* and proteins (proteoglycans and collagen type II) between caALK5- or caALK1-transduced pellets cultured in the absence of TGFβ and the control condition (LacZ-transduced pellets stimulated with TGFβ). Compared to the LacZ control stimulated with TGFβ, caALK5-transduced pellets expressed *ACAN* 99.6% lower (Fig 4A) and *COL2A1* 99.9% lower (Fig 4B). In caALK1-transduced pellets *ACAN* was 92.7% (Fig 4A) and *COL2A1* was 99.6% (Fig 4B) lower expressed than in LacZ-transduced pellets exposed to TGFβ. Consistent with these findings, caALK5-transduced pellets did not stain positive for proteoglycans (Fig 4C; upper panel) or collagen type II (Fig 4C; lower panel), and caALK1-transduced pellets stained weakly positive for the two cartilage-specific proteins. In contrast, LacZ-transduced pellets displayed intense staining of proteoglycans and collagen type II after 7 days stimulation with TGFβ (Fig 4C), confirming that only in this condition BMSCs differentiated and deposited a high amount of cartilage matrix. Taken together, these data indicate that neither constitutively active ALK5 nor constitutively active ALK1 induce chondrogenic differentiation of BMSCs as efficient as TGFβ.

### Downregulation of either ALK5 or ALK1 expression in BMSCs inhibits TGFβ-induced chondrogenic differentiation

To further determine the role of ALK5 and ALK1 receptors in chondrogenesis initiated by TGFβ, we investigated whether downregulation of either ALK5 or ALK1 expression abrogated TGFβ-induced chondrogenic differentiation of BMSCs. ALK5 and ALK1 were downregulated by transducing human fetal BMSCs, cultured in monolayer, with a lentivirus overexpressing either ALK5 short hairpin RNA (ALK5-shRNA) or ALK1-shRNA. Following transduction, BMSCs were pellet-cultured in chondrogenic medium.

**Fig 5:**
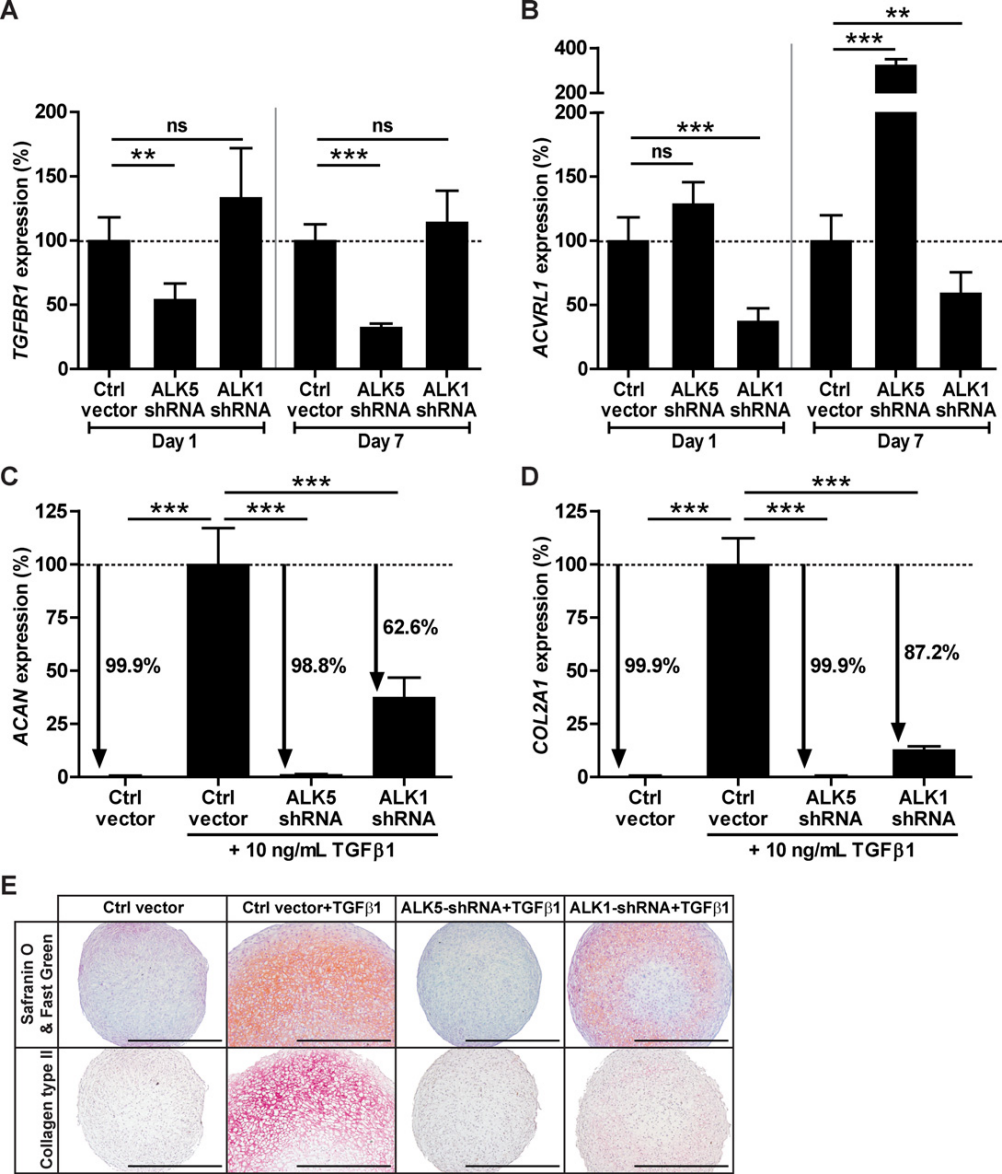
shRNA-mediated downregulation of either ALK5 or ALK1 inhibits TGFβ-induced chondrogenic differentiation of BMSCs. Human fetal BMSCs (donor F1) were transduced with lentiviral ALK5-shRNA, ALK1-shRNA or empty vector as control (Ctrl vector) and pellet-cultured in chondrogenic medium containing TGFβ. Short hairpin RNA-mediated downregulation of ALK5 and ALK1 was determined by gene expression of *TGFBR1* **(A)** and *ACVRL1* **(B)** in pellets cultured for 1 or 7 days. The effect of ALK5-shRNA and ALK1-shRNA on TGFβ-induced chondrogenesis was evaluated by transcript analysis of *ACAN* **(C)** and *COL2A1* **(D)** and by staining proteoglycans with Safranin O/Fast Green **(E; upper panel)** and collagen type II with immunohistochemistry **(E; lower panel)** in pellets cultured for 7 days. Representative images of consecutive pellet sections per condition are shown and the scale bar represents 500 μm. Gene expression was normalized to reference gene *RPS27a* and data are expressed as % relative to normalized mRNA levels in control vector-transduced pellets stimulated with TGFβ. Bars represent mean ± SD of quadruplet pellets from 1 representative experiment (out of 3), ns = not significant; **p<0.01; ***p<0.001.

At day 1 and 7 of chondrogenic induction we verified the efficiency of shRNA-mediated downregulation by gene expression analysis of *TGFBR1* and *ACVRL1*. Compared to the control condition at both time points, *TGFBR1* expression was about 50% downregulated by ALK5-shRNA (Fig 5A) and *ACVRL1* expression was about 50% downregulated by ALK1-shRNA (Fig 5B). In addition, *TGFBR1* mRNA levels were not affected by ALK1-shRNA (Fig 5A), but *ACVRL1* was higher expressed in the ALK5-shRNA condition than in the control condition at day 7 (Fig 5B).The effect of shRNA-mediated ALK5 and ALK1 downregulation on TGFβ-induced chondrogenic differentiation of BMSCs was determined by *ACAN* and *COL2A1* gene expression analysis and examining deposition of proteoglycans and collagen type II in pellets stimulated with TGFβ for 7 days. Compared to control vector-transduced pellets stimulated with TGFβ, ALK5-shRNA reduced expression levels of *ACAN* by 98.8% (Fig 5C) and *COL2A1* by 99.9% (Fig 5D), resulting in the same low *ACAN* and *COL2A1* mRNA levels as in the unstimulated control condition. ALK1-shRNA reduced *ACAN* expression by 62.6% (Fig 5C) and *COL2A1* expression by 87.2% (Fig 5D) compared with the TGFβ-stimulated control condition. Furthermore, we observed that control vector-transduced pellets exposed to TGFβ stained strongly positive for proteoglycans (Fig 5E; upper panel) and collagen type II (Fig 5E; lower panel), whereas TGFβ-stimulated pellets overexpressing ALK1-shRNA were only weakly positive for both cartilage matrix molecules. Moreover, pellets consisting of ALK5-shRNA-transduced BMSCs did not stain positive for proteoglycans nor for collagen type II, despite stimulation with TGFβ (Fig 5E). Thus, shRNA-mediated downregulation of ALK5 or ALK1 results in an abrogation of TGFβ-induced chondrogenic differentiation of BMSCs.

## Discussion

In this study we investigated the involvement of ALK5 and ALK1 receptors in TGFβ-induced chondrogenic differentiation of BMSCs. We show that TGFβ modulated gene expression of its receptors in BMSCs by increasing ALK5 whilst decreasing ALK1, although mRNA levels of ALK5 and ALK1 did not associate with the chondrogenic differentiation capacity. Overexpression of either caALK5 or caALK1 receptors did not induce chondrogenesis as efficiently as that induced by TGFβ, and downregulating either ALK5 or ALK1 blocked cartilage formation in TGFβ-stimulated BMSCs. Taken together, these findings indicate that both ALK5 and ALK1 are required for TGFβ-induced chondrogenic differentiation of human BMSCs.

Although TGFβ is well-known to induce chondrogenic differentiation, no study has determined whether TGFβ induces chondrogenesis through activation of its ALK5 or ALK1 receptor. Therefore, in the present study we overexpressed either constitutively active ALK5 or constitutively active ALK1 and downregulated mRNA expression of the two TGFβ receptors in BMSCs. The characteristic of constitutively active ALK receptors is that they phosphorylate downstream SMAD proteins without prior activation by TGFβ, which allowed us to determine whether TGFβ induces chondrogenic differentiation through activation of its ALK5 or ALK1 receptor. Despite ALK5 is considered to be the 'classical' TGFβ receptor, overexpression of caALK5 did not induce chondrogenesis. Previously, caALK1 overexpression was reported to fail to promote cartilage nodule formation in ATDC5 cells (chondrogenic mouse teratocarcinoma cell line) [35]. Here, we demonstrate that caALK1 overexpression slightly induced cartilage matrix formation in BMSCs, albeit not comparable to TGFβ-induced matrix formation. Together these results suggest that, to induce chondrogenesis, activation of either ALK5 or ALK1 is insufficient. Since the abundance and timespan of SMAD phosphorylation has been demonstrated to influence the cell’s responses [36, 37], one has to take into account that SMAD phosphorylation might be initiated with different kinetics by exogenous TGFβ than by overexpression of constitutively active receptors.

As neither caALK5 nor caALK1 overexpression induced chondrogenic differentiation in single overexpression experiments, this suggests that both receptors are required to induce chondrogenesis. When co-transducing cells with two constructs, we cannot ensure double targeting of all single cells which is crucial for drawing conclusions on the impact of dual receptor overexpression on chondrogenesis. Instead, we determined the requirement of both receptors for chondrogenic differentiation by downregulating either ALK5 or ALK1 expression with short hairpin RNA. We show that knocking down either one of the two TGFβ receptors prevented chondrogenic induction by TGFβ, confirming that without the presence of both receptors chondrogenesis will not occur. Similar to our observation when we specifically downregulated ALK5, also inhibition of ALK5 with a small molecule (ALK5i) was reported to prevent chondrogenesis [38]. Although small molecule inhibitors might have off-target effects [39], the results of Elkasrawy *et al*. [38] and our study prove a crucial role for ALK5 in chondrogenic differentiation of BMSCs. Additionally, the importance of ALK5 in chondrogenesis is supported *in vivo* as mice with conditional knockout of ALK5 in skeletal progenitor cells had impaired endochondral bone formation; a process wherein mesenchymal stem cells first differentiate into chondrocytes which then terminally differentiate and eventually are replaced by bone cells [40]. Regarding the involvement of ALK1 in chondrogenesis, overexpression of kinase-inactive ALK1 receptors was previously found to block spontaneous cartilage nodule formation in a murine chondrogenic cell line [35]. We show here that shRNA-mediated downregulation of ALK1 in human BMSCs strongly reduced TGFβ-induced chondrogenic differentiation, further supporting that besides ALK5 also ALK1 is required for chondrogenesis.

To the best of our knowledge, this study is first to demonstrate ALK5 and ALK1 gene expression in *in vitro-*expanded human BMSCs as well as in chondrogenically differentiating human BMSC pellet cultures. We show that TGFβ, besides inducing chondrogenic differentiation of BMSCs, regulated expression of its receptors by enhancing ALK5 expression whilst downregulating ALK1 expression, skewing the balance between its two receptors towards ALK5. This is in line with previous reports demonstrating that TGFβ enhanced ALK5 gene expression in human smooth muscle cells, pancreatic epithelial cells and lung fibroblasts [41–43]. However, also the opposite was shown as TGFβ downregulated ALK5 mRNA and protein levels in human articular chondrocytes [44], suggesting that the effects of TGFβ on ALK5 expression might be culture condition- or cell type-dependent. So far, the effect of TGFβ on ALK1 was investigated in only two studies. Whereas TGFβ did not change ALK1 expression in neurons [45], TGFβ reduced ALK1 mRNA levels in human endothelial cells, which is similar to our observation in human BMSCs [46]. Moreover, in line with the observed effect of TGFβ on ALK1 receptor expression, we also demonstrate that caALK5 overexpression reduced ALK1 mRNA levels whereas downregulation of ALK5 enhanced ALK1 gene expression. In addition to a change in balance between ALK5 and ALK1 receptor levels by TGFβ, we show that the mRNA levels of ALK5 and ALK1 varied between different BMSC donors during onset of chondrogenesis. Previously, ALK5 gene expression was shown by us to correlate with expression of *ACAN* and *COL2* in human OA cartilage [24]. Although the chondrogenic capacity of different BMSC donors was diverse in our study, we could not find an association between ALK5 or ALK1 gene expression levels and the chondrogenic differentiation capacity of BMSCs. Since both TGFβ receptors appeared necessary for chondrogenesis of BMSCs, the importance of balance, timing of expression and activity of ALK5 and ALK1 deserves more attention in future studies.

In our study we used human BMSCs from both adult and fetal origin. Recently, Brady *et al*. found that TGFβ3 could induce chondrogenesis in adult BMSC pellets, whereas TGFβ3 as well as TGFβ1 did not initiate chondrogenic differentiation of fetal BMSC pellet cultures [47]. The authors suggested that, apparently, adult and fetal BMSCs use different signaling mechanisms for inducing chondrogenic differentiation. In our experiments, however, both adult and fetal BMSC pellets did form cartilage matrix molecules following stimulation with TGFβ1 and the mRNA levels of ALK5 and ALK1 as well as their response to TGFβ1 was similar. Because our observation is not in line with the findings from Brady *et al*., further investigation is necessary to exactly determine the dependency of chondrogenesis on TGFβ and whether this is related to the source and developmental stage of human BMSCs.

Our study provides new insights regarding the involvement of ALK5 and ALK1 in chondrogenesis induced by TGFβ. This new knowledge helps to better understand the molecular events that control chondrogenic differentiation of mesenchymal stem cells, which is important for developing procedures to optimize articular cartilage repair.
